# Synthesis and characterization of iron oxide-commercial activated carbon nanocomposite for removal of hexavalent chromium (Cr^6+^) ions and Mordant Violet 40 (MV40) dye

**DOI:** 10.1038/s41598-024-51587-6

**Published:** 2024-01-12

**Authors:** Soha Mahrous Ismail Mohamed, Murat Yılmaz, Eda Keleş Güner, Ahmed El Nemr

**Affiliations:** 1 Institute of Graduate Studies and Research, Department of Environmental Studies, Alexandria University, Alexandria, Egypt; 2 Bahçe Vocational School, Department of Chemistry and Chemical Processing Technologies, Osmaniye Korkut Ata University, Osmaniye, 80000 Türkiye; 3 Uzumlu Vocational School, Department of Property and Security, Erzincan Binali Yıldırım University, Erzincan, Türkiye; 4 National Institute of Oceanography and Fisheries (NIOF), Kayet Bey, Elanfoushy, Alexandria Egypt

**Keywords:** Environmental chemistry, Pollution remediation

## Abstract

Iron Oxide-commercial activated carbon nanocomposite (CAC-IO) was prepared from commercial activated carbon (CAC) by the co-precipitation method, and the resulting nanocomposite was used as an adsorbent to remove hexavalent chromium (Cr^6+^) ions and Mordant Violet 40 (MV40) dye from wastewater. The produced materials (CAC, CAC after oxidation, and CAC-IO) were comparatively characterized using FTIR, BET, SEM, EDX TEM, VSM, and XRD techniques. The adsorption mechanism of Cr^6+^ ions and MV40 dye on CAC-IO was examined using Langmuir and Freundlich isotherm models.. Different models were applied to know the adsorption mechanism and it was obtained that Pseudo-second order fits the experimental data better. This means that the adsorption of the adsorbate on the nanocomposite was chemisorption. The maximum removal percent of Cr^6+^ ions by CAC-IO nanocomposite was 98.6% determined as 2 g L^–1^ adsorbent concentration, 100 mg L^–1^ initial pollutant concentration, solution pH = 1.6, the contact time was 3 h and the temperature was room temperature. The maximum removal percentage of Mordant Violet 40 dye (C.I. 14,745) from its solutions by CAC-IO nanocomposite was 99.92% in 100 mg L^–1^ of initial dye concentrations, 1.0 g L^–1^ of adsorbent concentration, solution pH = 2.07, the contact time was 3 h. The MV40 dye adsorption on CAC-IO was the most fitted to the Freundlich isotherm model. The maximum adsorption capacity was calculated according to the Langmuir model as 833.3 mg g^–1^ at 2 g L^–1^ of adsorbent concentration and 400 mg L^–1^ of initial MV40 dye concentration. The Cr^6+^ ions adsorption on CAC-IO was more fitted to the Freundlich model with *Q*_*max*_, equal to 312.50 mg g^–1^ at 1 g L^–1^ adsorbent concentration and 400 mg L^–1^ of Cr^6+^ ions initial concentrations.

## Introduction

The rapid development of technology, excessive consumption demand of people, irregular urbanization, uncontrolled population growth, developments in the industrial field, nuclear waste, and heavy metal accumulation cause pollution of natural resources and constitute the most important environmental problems of today. Dyestuffs, heavy metals, organic pollutants, radioactive pollutants, highly toxic substances, and pharmaceutical chemicals are the contents of industrial wastes that cause serious environmental problems^1–4^.

Dyes, heavy metals, and other waste materials used in many branches of industry, especially textiles, cosmetics, paper, pharmaceuticals, plastics, dye and leather threaten the environment by polluting water resources^5–8^. The textile industry uses a variety of dyes, including basic, acid, reactive, direct, azo, mordant, vat, and dispersion dyes^9^. Because they have the greatest color variation, the largest volumes, and the most adaptable properties of all the synthetic dyes, azo dyes take the top spot. Mordant Violet 40 dye is an example of a dye containing only one azo group, this dye can be found in the aquatic environment, is toxic and mutagenic to the ecosystem, may cause harmful effects on organisms, the effect relies upon the exposure time of the azo dye to the dye in water, as well as its concentration in water^9,10^.

Treatment of wastewater containing heavy metals is still a difficult and serious environmental concern. One of the heavy metals whose compounds are extensively employed in the chemical industry is chromium^11^. Some of the other application areas where chromium is used are the electroplating, textile, leather tanning, metal plating, production of organic chemicals, wood preservation, and chromate preparation industries^12–15^. Hexavalent chromium and trivalent chromium are most common in the environment, also chromium possesses several oxidation states (from – 2 to + 6). Cr^6+^ is highly soluble in water and bioavailable, while Cr^3+^ is less soluble and stable^16^. The Cr^6+^ ions, which is found to be toxic to stem cells and living organisms, harms human health because it is highly mutagenic and carcinogenic. Since chromium compounds are widely used and accumulated in natural environments, it is very important and necessary to remove them from the environment^17^.

Methods such as chemical precipitation, membrane filtration, electrochemical treatment, and adsorption are some of the traditional wastewater treatment technologies applied to remove heavy metals and dyestuffs from the water environment^18–22^. Studies to discover new techniques in the field of environmental technology have been guided by the shortcomings, application challenges, or costs of current treatment approaches. Among the mentioned treatment technologies, adsorption is the most efficient in terms of development and applicability^23,24^. The cost-effectiveness of the adsorption method, its ease of use, the easy separation of toxic organic–inorganic species from the aqueous environment, and the disposal of the separated toxic material without affecting the biological structures highlight the advantages of the method^25,26^.

Iron oxide (Fe_3_O_4_), titanium dioxide (TiO_2_), aluminum oxide (Al_2_O_3_), copper oxide (CuO), zinc oxide (ZnO), and nickel oxide (NiO) nanomaterials are well-known metal-based nano adsorbents for heavy metal removal and dyestuff^27–30^. By reducing the particle size, metal oxide nanoparticles were able to absorb more substances. To remove metal and organic pollutants simultaneously, metal oxide nanoparticles are injected into the skeleton of activated carbon or other porous materials^31^. By altering the pH of the fluid, it is also possible to recreate these metal-based nanoparticles, and after a few regenerations, they are sufficiently potent^32^. Some of these metal oxides are iron oxide nanoparticles such as magnetite (Fe_3_O_4_), maghemite, hematite (*α*-Fe_2_O_3_), and akaganéite (*β*-FeOOH) were used as nano-sized adsorbents to remove some of heavy metals and dyes from water^33–35^. Magnetic nanoparticles are becoming attractive for industrial and environmental applications due to their low cost, lossless recovery, and reusability^36,37^. However, aggregation of magnetite nanoparticles in water is undesirable, which reduces their catalytic capabilities. Various methods have been developed to overcome the coagulation of nanoparticles. In order to overcome this limitation and increase the catalytic activity of magnetic nanoparticles, materials such as graphene, graphene oxide, activated carbon, silica, carbon nanotube, bentonite, kaolinite, montmorillonite, and zeolite, etc. with high adsorption ability and catalytic activity are used as solid supports^38–46^. Thanks to the use of these materials as a support, the surface area of iron-based magnetic nanoparticles is increased and their distribution is facilitated. At the same time, nanomagnetic particles placed on these support materials have smaller particle sizes, less agglomeration, and higher thermal and chemical stability than those without support materials^47,48^.

Due to its high surface area and pores, activated carbon has a great potential to adsorb organic and inorganic compounds in the liquid and gas phases^49^. Since activated carbon has a large surface area, it has a great affinity for absorbing many deformations. AC is one of the most effective, economical, and simplest adsorbents for pollutants in aqueous solutions^50,51^. Although it can be found in organic cells as an adsorbent, activated carbon is difficult to extract from its solution. Electric or magnetic fields cannot regulate activated carbon because it lacks polarity. It is aimed to overcome this problem by synthesizing many activated carbon composite adsorbents with magnetic properties^52–54^. Technology for treating water might potentially develop with the addition of magnetic characteristics to activated carbon. Adsorption and magnetic separation work in concert to offer a flexible and effective solution to problems with water quality, all the while enhancing the overall sustainability of water treatment procedures. The goal of this field's ongoing research and development is to improve and broaden the uses of magnetic activated carbon in the pursuit of more sustainable and clean water sources^10^. The magnetic property of activated carbon is introduced through the incorporation of magnetic nanoparticles, such as magnetite (Fe_3_O_4_). This property allows for easy separation of the adsorbent from the solution after adsorption. Magnetic activated carbon can be manipulated using an external magnetic field, making the process more efficient and environmentally friendly^9,10^.

In this study, iron oxide nanomagnetic composite (CAC-IO) was prepared from fisher commercial activated carbon (CAC) by co-precipitation method. This work is the first to use this type of commercial activated carbon to produce magnetic activated carbon composite and use it in the adsorption of heavy metals and textile dyes. The CAC-IO was invesrigated as an adsorbent for toxic metal (Cr^6+^) and azo dye (Mordant Violet 40). However, to our knowledge, Mordant Violet 40 dye removal has only been published by us. The importance of CAC-IO is that it shows a high *Q*_m_ for the MV40 dye and Cr^6+^ ions compared to the published data. The nano-magnetic composite was synthesized by using different iron salts and different base solutions. The magnetic iron oxide nanocomposite was comparatively characterized by FTIR, BET, SEM, EDX TEM, VSM, and XRD techniques. Different adsorption and kinetic isotherm models were used to investigate the mechanism of adsorption of two pollutants Cr^6+^ions and Mordant Violet 40 (MV40) dye on the synthesized iron oxide nanocomposite (CAC-IO). The adsorption capacity of the CAC-IO nanocomposite was calculated using the Langmuir and Freundlich models. To ascertain the adsorption mechanism, pseudo-first-order (PFO), pseudo-second-order (PSO), and intraparticle diffusion (IPDM) models were applied.

## Materials and methods

### Materials

Activated charcoal powder was purchased from Fisher Scientific, UK. Iron (III) Nitrate Nona hydrate (98%), and Iron (II) chloride hydrate were obtained from LOBA Chemie Company, India. Ferrous Sulphate Heptahydrate (FeSO_4_.7H_2_O), Ferric chloride (FeCl_3_), Ammonia solution (25%), Sodium hydroxide, Sodium carbonate and Ethanol were obtained from El Nasr Company, Egypt. Hydrogen peroxide (50%) was purchased from Gateway Company, Hydrochloric acid solution (37%) and Sulfuric acids (98%) were purchased from Merck Company; Potassium dichromate was purchased from Sigma Aldrich Company, USA. MV40 dye salt was purchased from ISMA dye Company, Kafer El Dwar, Egypt.

### Surface modification of commercial activated charcoal (CAC)

CAC oxidation was performed to increase the active site and functional group on the surface of CAC to obtain better contact with iron oxide. Oxidation of commercial activated charcoal powder was achieved by placed of 100 g of CAC in 1800 mL of hydrogen peroxide solution (8%) in the presence of ozone flow for 2 h. The carbon suspension was then filtered with a vacuum. After sequential pumping, several times distilled water and ethanol were used, respectively, for washing until the pH stabilized (approximately neutral). The resulting wet powder was dried in an oven (105 °C) for 24 hto remove its moisture and then weighed to obtain 91 g of dried powder^55,56^.

### Preparation of ıron oxide-commercial activated carbon nanocomposite (CAC-IO)

Co-precipitation method was used to create an iron oxide nanocomposite from commercial activated carbon by dissolving 4.04 g of iron (III) nitrate nonahydrate (98%) and 1.2 g of iron (II) chloride hydrate in 500 mL of distilled water in 1000 mL added to 10 g of CAC powder in a round table flask (3.38:1:8.33 of Fe(NO_3_)_3_:FeCl_2_:oxidized CAC, respectively)^57^. The obtained suspension was ultrasonically agitated in a sonicator at ambient temperature and normal atmospheric pressure for 30 min. The ultimate pH was 13.72 after adding 100 mL of sodium hydroxide solution (5 M) drop by drop over the course of 45 min. The flask containing the created composite was moved to be refluxed for 16 h at moderate temperatures after the base solution had been fully added. After cooling to ambient temperature, the iron oxide nanocomposite from commercial activated carbon was filtered and collected with a magnet. It was then repeatedly cleaned with distilled water before being exposed to 98% ethanol. The nanocomposite was dried in an electric oven, and the 11.63 g of CAC-IO powder were measured using a balance.

### Characterization

The characterisation of CAC-IO nanocomposites has involved the application of a number of approaches. The surface functional group on the CAC-IO powder and CAC after treatment was identified using an FT-IR Spectrophotometer with an ATR unit. The samples' ATR-FTIR spectra were taken using a Bruker VERTEX 70 spectrophotometer in the range of 4000 to 400 cm^-1^. The produced samples (CAC after treatment, CAC-IO) were measured using Nitrogen-adsorption isotherm to determine their surface area, pore volume, and pore size distribution. At 77 K, sample measurements were started after the pressure was brought to *P*/*P*_*o*_ = 0.99. Using the Belsorp Mini II, Version 1.2.5 surface area analyzer, the average pore diameters and total surface areas of the samples were calculated using the Brunauer, Emmett, and Teller (BET) equation. The surface morphology and porosity of CAC after treatment and CAC-IO samples were examined using an analytical Scanning Electron Microscope (JEOL JSM-6360LA). After the samples were powdered, they were coated with a gold layer to obtain clearer images and increase conductivity.

The morphology and particle size of the CAC-IO sample were examined using ESL Transition Electron microscopy from Scientific Researches City. For this purpose, 2 mg of the powdered samples was taken and dissolved in 5 mL of ethanol and mixed in the centrifuge device. A drop of the resulting suspension was tested by dropping it onto a copper grid. The degree of crystallinity and phase compositions of prepared samples were determined by an X-ray diffraction device (model No, 202,964) from Beni Sweif University. The Cu-Kα radiation was used to generate the XRD pattern at 10 mA, 1.54Å wavelength, and 25 °C in the 2θ region of 10–80°.

The magnetic property of the nanocomposite was realized with the VSM device at Beni Sweif University. It ranged from + 20 KOe to –20 KOe for the magnetic field of G. The amount of MV40 dye in aqueous solutions was measured using an Analyticjena Spekol 1300 UV–VIS Spectrophotometer (Model No. 4560002, Cole Parmer Instrument Co., USA).

### Adsorption experiments

Separately, a volumetric flask was used to dissolve a specific quantity of K_2_Cr_2_O_7_ and MV40 dye salts in 1000 mL of distilled water to create a stock solution of 1000 mg L^–1^ of Cr^6+^ solution and MV40 dye. The stock solutions for the Cr^6+^ ions and MV40 dyes were produced separately from their diluted concentrations. The different Cr^6+^ ion concentrations and MV40 dye solution concentrations (100, 150, 200, 300, and 400 ppm) were added separately to the adsorption batches along with various concentrations of CAC-IO composite (1.0, 1.5, 2.0, and 2.5 g L^–1^). Each concentration had a volume of 100 mL in a conical flask, and the adsorbent-adsorbate suspensions were agitated using a shaker at room temperature and 200 rpm for 180 min for each pollutant's specific equilibrium duration. To determine the amount of leftover Cr^6+^ ions and MV40 dye in each solution, a sample of each solution was obtained at regular intervals. In adsorption experiments, it took 3 h for the samples to reach equilibrium, and analyses were performed at the end of this period. Adsorption tests were carried out with 0.5 ml Cr^6+^ ions sample or 2 ml MV40 dye sample at intervals of 5, 10, 20, 30, 45, 60, 90, 120, 150, and 180 min. Then, the composites were separated from the solutions by centrifugation at 6000 *rpm* for 5 min, and a magnet was used to disperse the composites in the solution and prevent the samples from separating. Cr^6+^ ion and MV40 dye filtrates obtained after centrifugation were measured at absorbance wavelengths of 540 and 510 nm, respectively^2,10,58^, on a spectrophotometer device for concentration determination. The effects of parameters such as initial concentration of adsorbates, nanocomposite concentration , contact time, and pH, which affect the removal of Cr^6+^ ions and MV40 dye from aqueous solutions of the prepared iron oxide nanocomposite CAC-IO samples were examined.

The experimental data from adsorption batches were tested by using different adsorption isotherm (Langmuir and Freundlich) and kinetic (PFO, PSO, IPDM) models. These models facilitated knowledge of the mechanism of adsorption in our study on nanocomposite adsorbent.

The removal % (R%) can be calculated by Eq. (1)^10^.

1 $$R\%=\frac{({C}_{0}-{C}_{t})}{{C}_{0}}x100$$

where *C*_*o*_ and *C*_*t*_ are the initial and final concentrations of adsorbate in aqueous solution, respectively. Adsorption capacity *q* (mg g^-1^) can be calculated from Eq. (2).

2 $$q=\frac{\left({C}_{0}-{C}_{t}\right)*m}{V}*100$$

where *m* is the mass of the iron oxide nanocomposite in grams and *V* is the volume of the adsorbate solution in Liter (L).

The pH of different solutions was measured at 1.0 g L^–1^ of adsorbent concentration (CAC-IO), 100 mL solution of 100 mg L^–1^ of Cr^6+^ ions, and MV40 dye concentrations individually for 3 h of contact time. The MV40 dye solutions and Cr^6+^ ion concentrations ranged in pH from very acidic to strongly basic solutions (pH = 1 to 11); the pH of the adsorbate solution under study was slightly different from this pH range.

## Results and discussion

### Characterization of adsorbent

#### FTIR analyses

**Figure 1:**
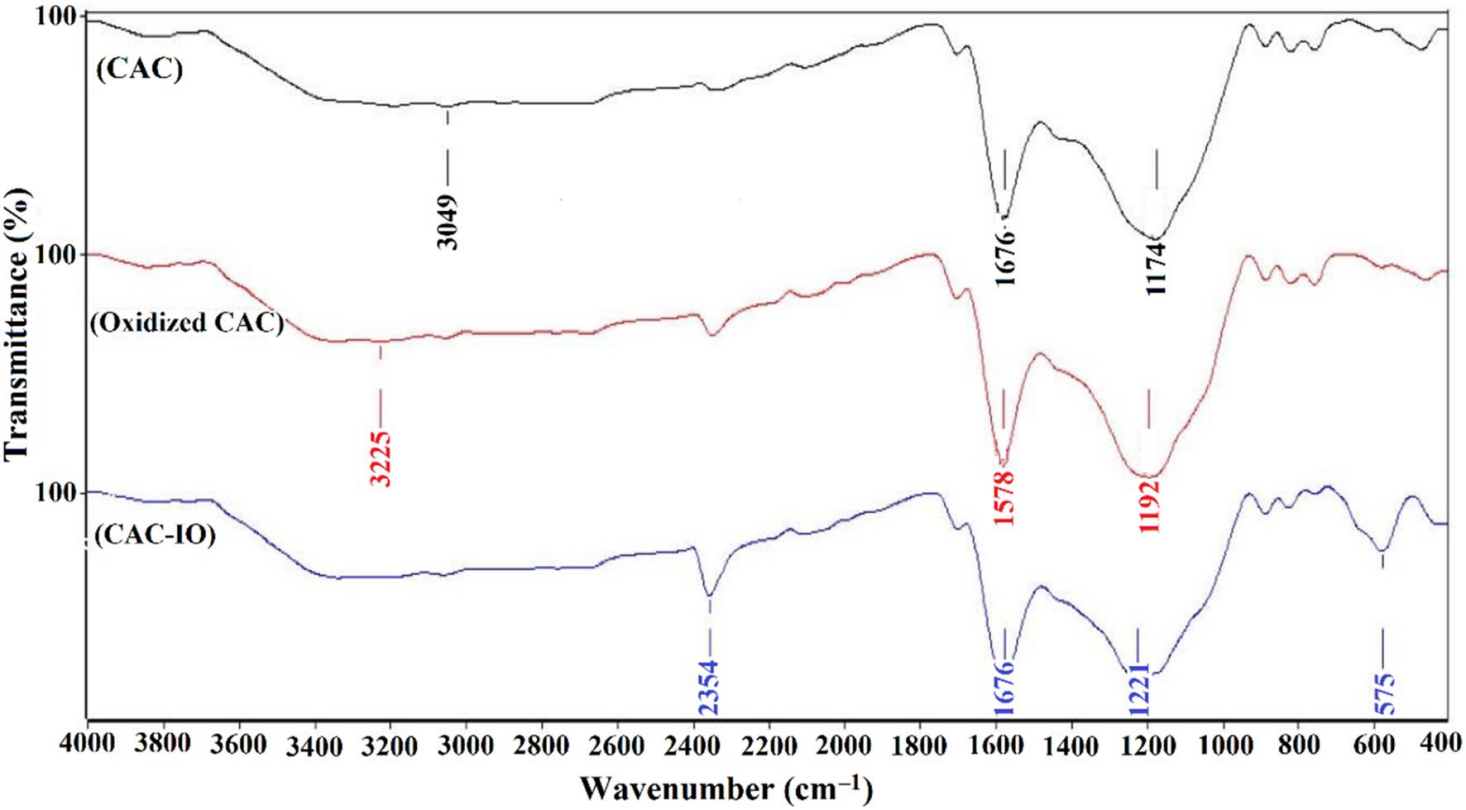
FTIR spectrum for CAC, CAC after oxidation and CAC-IO nanocomposite.

The produced materials (CAC, CAC after treatment, and CAC-IO) were characterized using the Fourier transform infrared technique, as shown in Fig. 1. The FTIR spectrum of CAC, CAC after oxidation, and CAC-IO nanocomposite showed a broad peak at 3049 and 3225 cm^–1^ due to the OH bond, also a peak appeared at 1576, 1578, 1576 cm^–1^ with small shifts due to the C=C stretching bond and the bands appeared at 1174, 1192 and 1221 cm^–1^ assigned to C–O stretching from phenolic, alcoholic, etheric groups and to C–C bond, similar results was obtained by Bagheri et al. (2017)^59^. The fact that all spectra included peaks at 2354 cm^–1^ caused by the C–C bond in the structure of activated carbon showed that the structure had not been damaged during the composite pyrolysis^53^. Due to the synthesis of nano-iron oxide, a new peak at 575 cm^–1^ in the CAC-IO spectra had been developed. The appearance of the Fe–O stretching bond revealed that iron oxide nanoparticles had been deposited on the CAC-IO adsorbent surface. The peaks appeared at 886 and 793 cm^–1^, which may be due to the δ(OH) and γ(OH) vibration in and out of the plane, respectively, indicated to geothite peaks^57,60–62^.

#### BET analyses

Using the BET equation, the surface area and pore information of the produced nanocomposite and its constituent material were calculated. The pore volume (*V*_*t*_) for each adsorbent and its precursor materials was calculated using nitrogen adsorption at relative pressure *P*/*P*_*o*_ = 0.99. The pore diameters of commercial and synthesized activated carbon and nanocomposites were calculated.

As seen in Table 1 using BET analyses, the specific surface areas of CAC, CAC after oxidation, and CAC-IO were calculated as 1426.8, 985.58, and 1070 m^2^ g^–1^, respectively. The use of H_2_O_2_, a powerful oxidizing chemical, caused damage to the pores in CAC's structure, resulting in a reduction in surface area following oxidation^55,63,64^. As the surface area increases after adding Fe_3_O_4_, it expected that the Fe_3_O_4_ is attached to the functional group on the surface of CAC rather than inserted inside the pores.

**Table 1:** Data of surface analyses of CAC, CAC after oxidation and CAC-IO.

Surface analyses	CAC	CAC after oxidation	CAC-IO
Pore diameter (nm)	3.3411	3.4575	3.4226
Pore volume (cm^3^ g^−1^)	1.1910	0.8519	0.9155
Surface area (m^2^ g^−1^)	1426	985.58	1070

**Figure 2:**
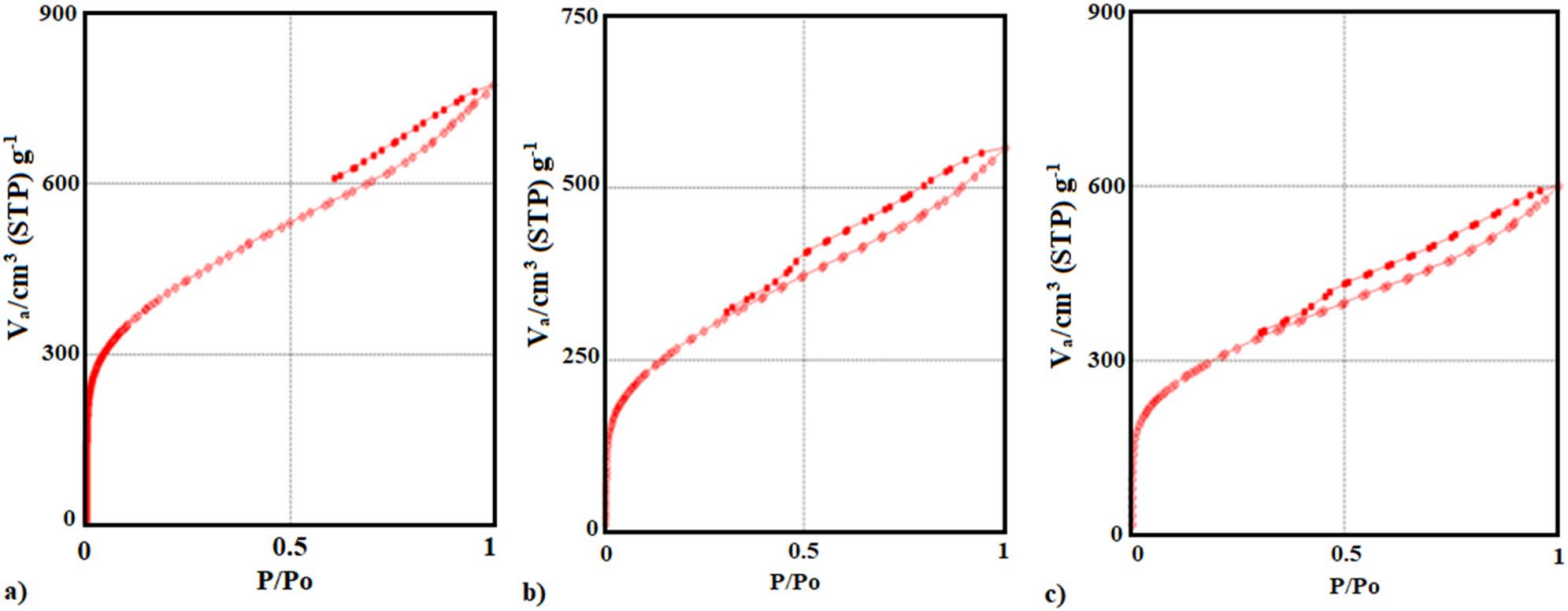
N_2_ adsorption–desorption isotherm plot of CAC (**a**), CAC after oxidation (**b**) and CAC-IO nanocomposite (**c**).

Figure 2 showed the nitrogen adsorption–desorption isotherms of the precursor materials and showed that they were type (IV) isotherms^65^. The structure of CAC and CAC after oxidation, as well as CAC-IO, were mesoporous^66^, according to the IUPAC classification, which is micropores (*d* < 2 nm), mesopores (2 < *d* < 50 nm), and macropores (*d* > 50 nm)^65^.

The CAC-IO nanomagnetic adsorbent's pore volume was 0.9155 cm^3^ g^–1^. The findings demonstrated that the oxidation process and the resulting production of magnetic nanocomposites reduced the surface area of the commercial activated carbon (CAC), which was caused by the dispersion of iron oxide nanoparticles on the carbon surface, from 1426 m^2^ g^–1^ to 1070 m^2^ g^–1^.

#### SEM-TEM analyses

**Figure 3:**
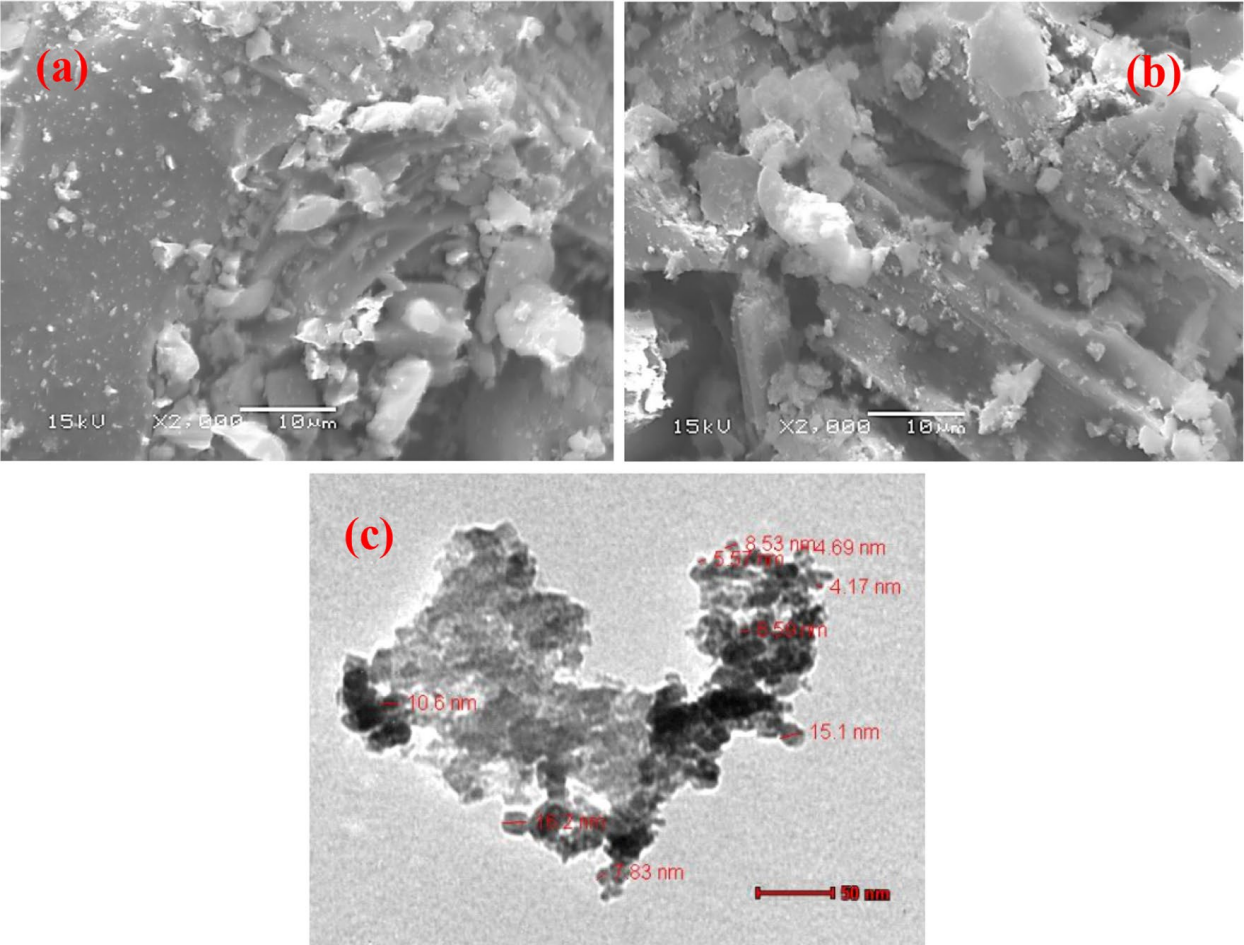
SEM Images of CAC after oxidation (**a**) and CAC-IO nanocomposite (**b**) at 15 kV and 2000 × magnification and TEM image of CAC-IO nanocomposite (**c**) at 50 nm scale.

As shown in Figs. 3a,b, scanning electron microscopy was used to examine the surface morphology and shape of the adsorbent prepared after oxidation and its iron oxide nanocomposite (CAC-IO). The surface of CAC-IO showed roughness than that of CAC, which may explain the attachment of Fe_3_O_4_ to the surface functional group instead of being inserted into the surface pores. The TEM image of the CAC-IO nanocomposite obtained using the Transition Electron Microscopy (TEM-2100 Electron Microscope) to determine the nano-sized composite spacing and their shape at the nanoscale is shown in Fig. 3c. TEM image of CAC-IO nanocomposite showed the particle shape of nano iron oxide was spherical and agglomerated to each other. According to Fig. 3c, the particle size ranged from 4.12 to 19.5 nm, and smaller particles have a higher adsorption capacity.

#### EDX analyses

**Figure 4:**
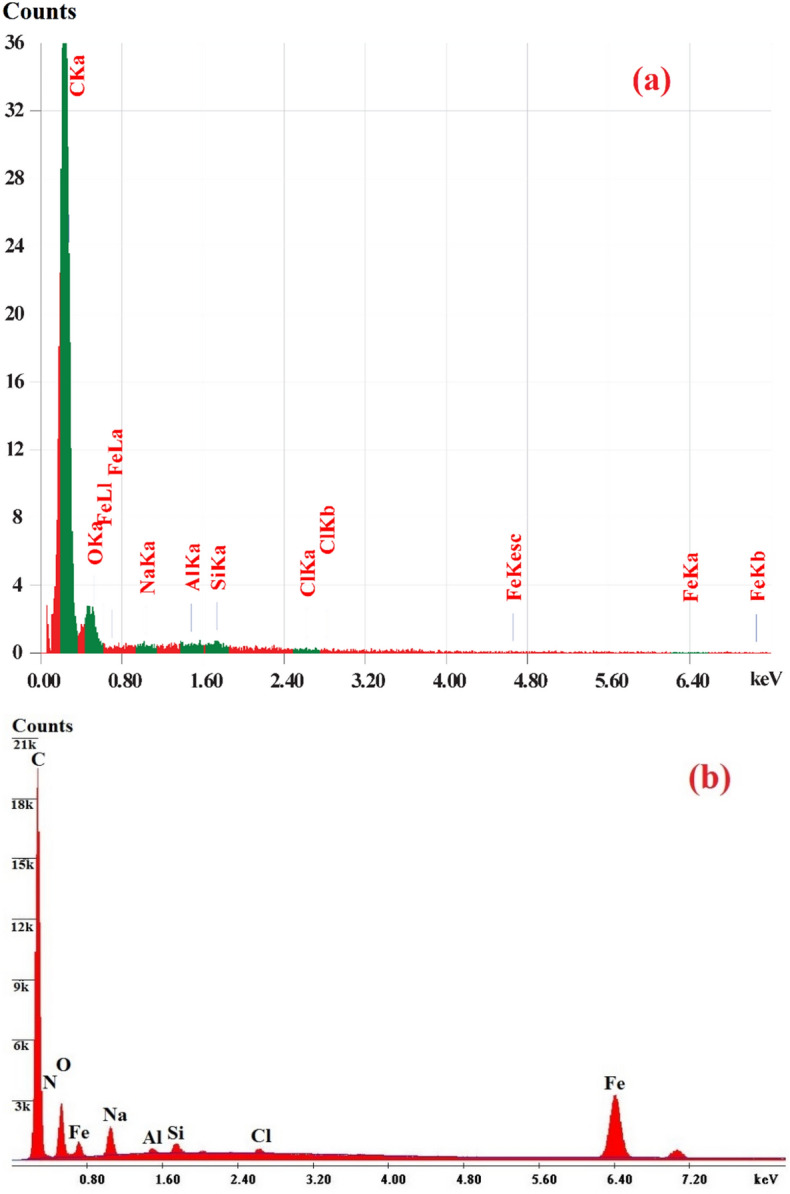
EDX analyses chart of CAC after oxidation (**a**) CAC-IO nanocomposite (**b**).

As shown in Table 2, the elements and iron oxide nanocomposites in the adsorbent produced during the treatment were identified and determined using the SEM–EDX equipment. The study of CAC after oxidation verified the existence of several components, including Carbon, Oxygen, Sodium, Silicon, and Chlorine with percentage ratios of 83.26, 15.38, 0.17, 1.05, and 0.14%, respectively, in the CAC structure. The iron element Fe has a weight ratio of 12.09% owing to the magnetic CAC-IO synthesis, and the examination of CAC-IO nanocomposites in Fig. 4 confirms the presence of the same components in CAC after oxidation with a modified weight ratio. The production of iron oxide nanocomposites with iron components resulted in a reduction in the carbon content from 83.26 to 65.25%^66^.

**Table 2:** The EDX of CAC after oxidation and CAC-IO nanocomposite.

Element	C	N	O	Na	Al	Si	Fe	Cl	Total
wt% of CAC after oxidation	83.26	–	15.38	0.17	–	1.05	–	0.14	100
wt% of CAC-IO nanocomposite	65.25	2.53	15.71	3.29	0.33	0.55	12.09	0.27	100

#### VSM analyses

**Figure 5:**
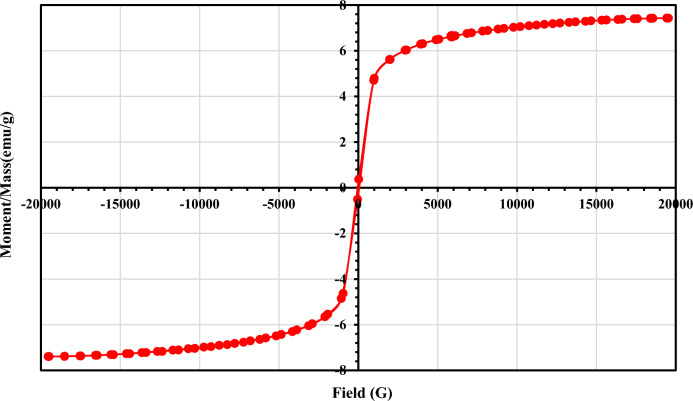
Magnetization curve for CAC-IO nanocomposite.

The magnetization curve of the synthesized magnetite iron oxide nanocomposite (CAC-IO) was measured to study the magnetic properties at room temperature in a magnetic field with a cycle of –20 to + 20 KOe. The highest saturation magnetization for CAC-IO was 7.4130 emu g^−1^ as in Fig. 5, which is due to the high iron oxide content of the CAC-IO nanocomposite. The magnetic properties of CAC-IO (7.4130 emu/g) compared to the magnetic properties of pure Fe_3_O_4_ nanoparticles (~ 90 emu/g) may be expained by the formation of CAC composites with Fe_3_O_4_ nanoparticles, which has a substantial impact on the magnetic properties of Fe_3_O_4_. This phenomenon can be attributed to factors such as the surface area and elemental composition of Fe_3_O_4_ within the composite or to the percentage of Fe_3_O_4_ within the CAC-IO composite.

#### XRD analyses

**Figure 6:**
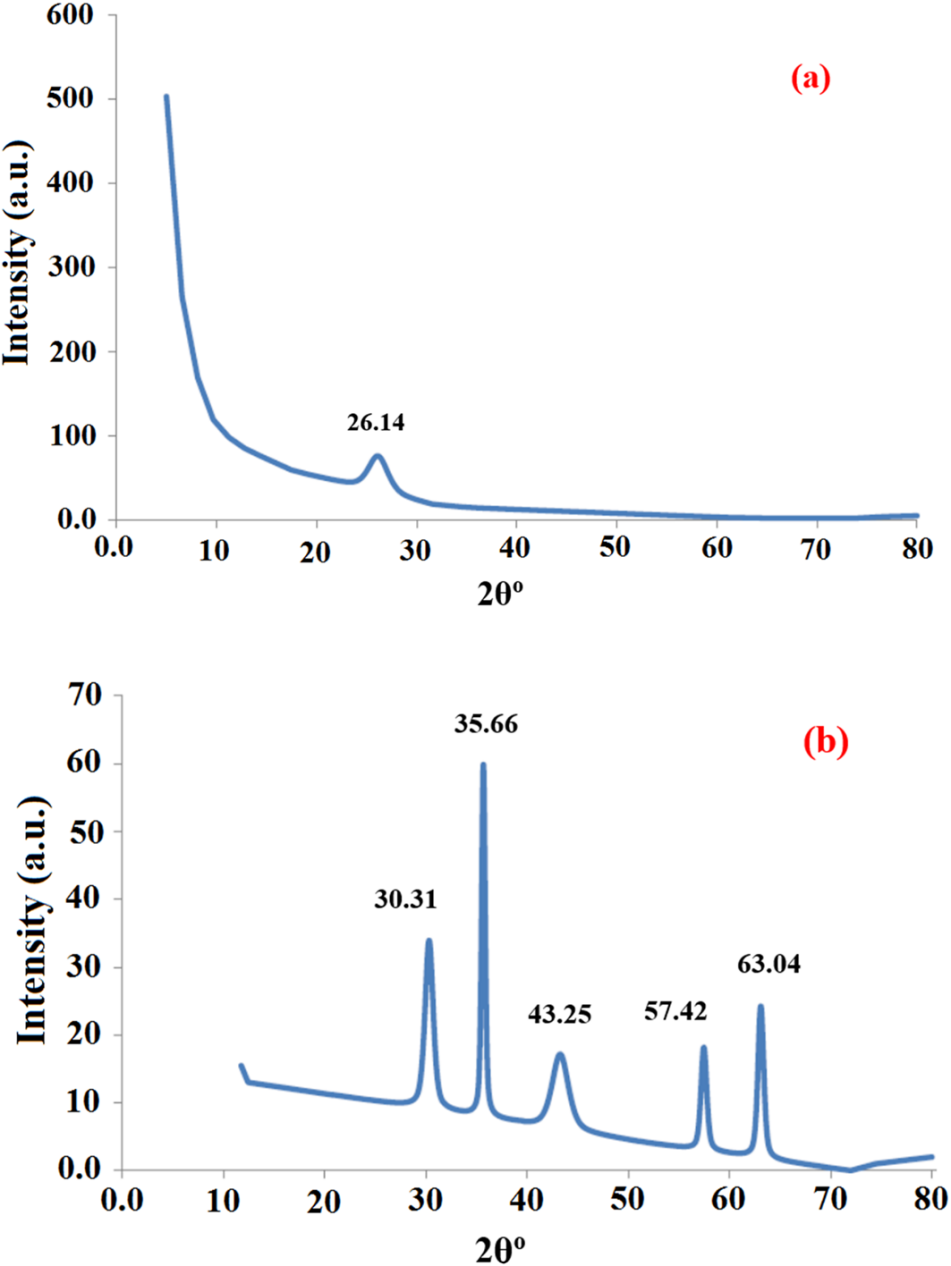
XRD graph of CAC after oxidation (**a**) and CAC-IO (**b**).

XRD analyses of the prepared nanocomposites and their pure materials obtained under Cu-kα radiation at 25 °C are given in Figs. 6a,b. In Fig. 6a, the only peak that appeared at 26.14° is related to commercial activated carbon after oxidation, similar results were obtained according to Gholamvaisi et al*.* (2014)^67^.

Figure 6b indicates to CAC-IO nanocomposite that shows several peaks at different angles at 30.31, 35.66, 43.25, 57.42, and 63.04°, the maximum peak intensity was at 2θ = 35.66^0^ which indicates the presence of magnetite (Fe_3_O_4_) or maghemite nanoparticles in cubic crystal structure shapes according to reference card code 04-013-9811. After oxidation, no peak for CAC was seen in Fig. 6b; this could be because the surface of the carbon has agglomerated or been coated with magnetite or maghemite nanoparticles^68^.

The average crystalline size of the prepared CAC-IO was determined from the XRD results; it was calculated from Scherrer’s formula (Eq. (3)),

3 $$ L = K\lambda /B{\text{Cos}}\theta , $$

where *L* is the crystalline size, λ is the wavelength of the X-ray, *B* is the full width of half maximum of a diffraction peak, *θ* is the angle of diffraction and *K* is the Scherrer’s constant of the order of 0.89^59^.

The XRD results show that the average crystal size of CAC-IO is 24.21 nm at 2θ = 35.66, which is in good agreement with the TEM results.

### Adsorption of Cr^6+^ ions and MV40 dye on CAC-IO nanocomposite

#### Effect of pH

**Figure 7:**
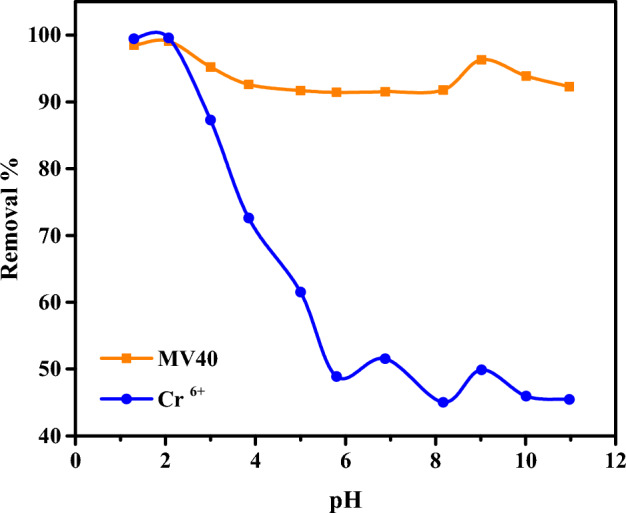
Effect of pH on the removal of Cr^6+^ ions and MV40 dye from aqueous media (Pollutant = 100 mg L^−1^, adsorbent = 100 mg L^–1^, Time = 3 h).

In order to examine the effect of solution pH on adsorption, solutions were prepared at constant concentrations at various pHs, and the adsorption of a certain amount of adsorbent and a certain volume of pollutant solution at room temperature was studied. As a result of the studies, the effect of different pH solutions has revealed that the best removal percentage is on acidic pH. Figure 7 shows that by increasing the pH of Cr^6+^ ions solutions from 1.6 to 11.24, the percentage of Cr^6+^ removal decreased from 99.58 to 45.45%, giving the maximum removal percentage at pH 1.6. The effect of pH value on the chromium species in the solution and its effect on the chromium removal % were previously studied^14,15,23,69–72^. pH Fig. 7 also shows that the removal % decreased by increasing the pH of MV40 dye solutions from 1.3 to 10.97, giving a maximum removal percentage was 99.08% at pH 2.07,100 mg L^–1^ of initial dye concentration with 1 g L^–1^ of CAC-IO concentration and 3 h of contact time. This result is due to the electrostatic attraction between the positively charged CAC-IO surface and the negative charges on the dye molecules at acidic pH, but at higher pH, there was repulsion between the two opposite charges of the dye molecules and the adsorbent surface used. Similar results were obtained in Kalantry et al. (2015)^73^.

#### Effect of CAC-IO adsorbent concentration

**Figure 8:**
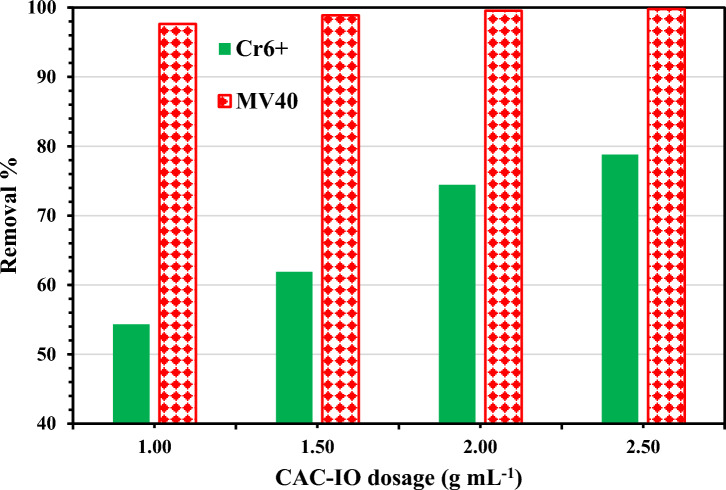
Effect of different CAC-IO concentrations on the removal % of Cr^6+^ ions and MV40 dye using 400 mg L^–1^ of pollutants' concentrations and 1.0 g L^–1^ as CAC-IO concentrations.

Different concentrations from CAC-IO nanocomposite (1, 1.5, 2, 2.5 g L^–1^) were used to study the adsorbent concentration effect at 400 mg L^–1^ of initial concentrations of Cr^6+^ ions and MV40 dye solutions, contact time is 3 h and fixed pH = 1.6 for Cr^6+^ ions solutions after adding the adsorbent while the dye solution pH was fixed at 2.07 for MV40 dye after adding the adsorbent separately. The resulting samples were drawn at intermittent times (10, 20, 30, 45, 60, 90, 120, 150, and 180 min) to separately analyze the final concentrations of Cr^6+^ ions and MV40 dye in solutions. Figure 8 illustrates the chart used to examine the influence of nanocomposite concentration on the percentages of Cr^6+^ ions and MV40 dye removal from water, respectively. The result shown for Cr^6+^ adsorption revealed that by increasing the adsorbent concentration (CAC-IO) from 1 to 2.5 g L^–1^, the removal percentage increased from 54.33 to 78.80%, so 2.5 g L^–1^ of CAC-IO nanocomposite was considered to be the optimum concentration to remove 400 mg L^–1^ of Cr^6+^ ions from aqueous media at optimum solution pH = 1.6, room temperature and equilibrium time = 3 h.

This results from the presence of unsaturated adsorption sites on adsorbent (CAC-IO) during the adsorption process, also the decrease in adsorption capacity may be due to the aggregation of adsorbent particles due to the high concentration of it. This aggregation may result in a reduction in the adsorbent's overall surface area and an increase in the diffusional route length^74^. The chart for the adsorption of MV40 dye indicated that increasing the adsorbent concentration (CAC-IO) from 1.0 to 2.5 g L^–1^ slightly enhanced the elimination percentage from 97.64 to 99.79%. At optimal solution pH = 2.07, room temperature, and equilibrium time is 3 h, 1.0 g L^–1^ of CAC-IO nanocomposite was thought to be the best concentration to remove 400 mg L^–1^ of MV40 dye from aqueous medium as shown in Fig. 8. However, there was no significant increase in the removal percentage of dye at concentrations larger than 1.0 g L^–1^.

#### Effect of initial adsorbate concentrations on CAC-IO nanocomposite

**Figure 9:**
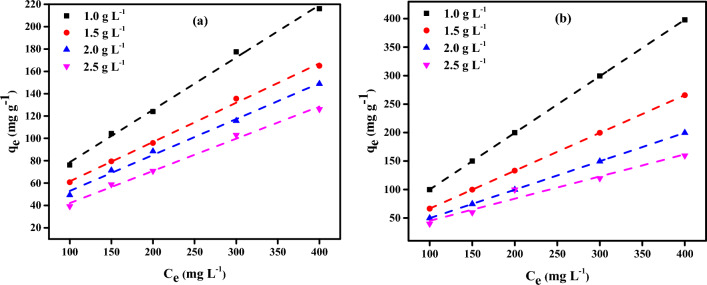
Effect of various concentrations of Cr^6+^ ions (**a**) and MV40 dye (**b**) on adsorption capacity *qe* (mg g^–1^) for each CAC-IO concentration (pH of Cr^6+^ ions solutions = 1.6 and pH of MV40 dye solutions = 2.07).

Different five concentrations (100, 150, 200, 300, 400 mgL^−1^) of Cr^6+^ ions and MV40 dye solutions were each examined during batch adsorption experiments at 1.0 g L^–1^ CAC-IO concentration individually at fixed pH = 1.6 in case of Cr^6+^ ions adsorption and pH = 2.07 in case of MV40 dye adsorption at room temperature. Commercial activated carbon-iron oxide nanocomposite (CAC-IO) was used to study the impact of initial Cr^6+^ ions concentrations on the rate of adsorption in the range of 100 to 400 mg L^–1^, as shown in Fig. 9a. It is obvious that the Cr^6+^ ions removal by different adsorbents doses (CAC-IO) was dependent on the initial Cr^6+^ ions concentrations, this is due to increasing the initial Cr^6+^ ions concentrations increased the amount of Cr^6+^ ions adsorbed on the adsorbent adsorption capacity (*q*_*e*_).

This increase is due to the resistance to the uptake of solute from Cr^6+^ ions solution decreased so the initial concentrations of Cr^6+^ solutions provide an important driving force to overcome the mass transfer resistance of Cr^6+^ ions between the aqueous and the solid phases^75^. In the range of 100 to 400 mg L^–1^, the impact of initial MV40 dye concentrations on the rate of adsorption by CAC-IO was examined, as shown in Fig. 9b. Additionally, the number of MV40 dye molecules adsorbed on the CAC-IO surface developed when initial dye concentrations were raised, which is why the MV40 dye removal by various adsorbent (CAC-IO) concentrations were reliant on those initial dye concentrations. As it was previously mentioned, this rise results from a reduction in the impedance to solute absorption from dye solution^75^.

#### Effect of contact time using CAC-IO

An experiment was done to study the effect of contact time, 100 mg L^–1^ of Cr^6+^ ions or MV40 dye initial concentrations were tested, and 2 g L^–1^ CAC-IO adsorbent concentration (highest dose gave maximum removal %) in case of Cr^6+^ ions solutions and 1.0 g L^–1^ CAC-IO adsorbent concentration in case of dye solutions at pH of Cr^6+^ ions and dye solutions, 1.6 and 2.07, respectively, and room temperature. The obtained samples were taken at interval times (10, 20, 30, 45, 60, 90, 120, 150, and 180 min) and analyzed by UV–visible spectrophotometer at 540 and 510 nm of maximum wavelengths of Cr^6+^ ions and MV40 dye, respectively.

**Figure 10:**
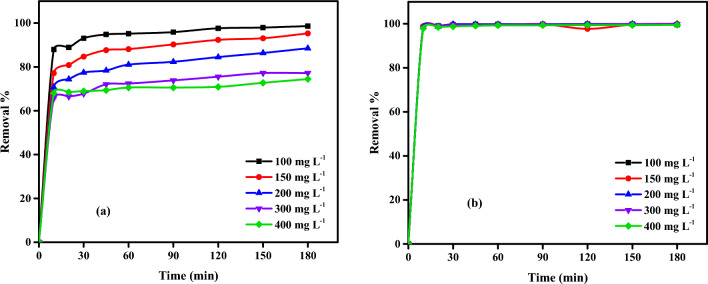
Effect of contact time on adsorption of Cr^6+^ ions (**a**) and MV40 dye (**b**) on CAC-IO nanocomposite at optimum dose = 2 g L^–1^ (pH of Cr^+6^ ions solutions = 1.6 and pH of MV40 dye solutions = 2.07).

The rapid removal of Cr^6+^ ions after only 10 min (87.92%) in the initial phase of adsorption from 0 to 10 min and then the rate of removal gradually slowed down until the equilibrium state was reached after 180 min, as shown in Fig. 10a. The rate of removal of MV40 dye was very fast (98.49%) from 0 to 10 min, and then the rate of removal gradually slowed down until it reached a constant value at equilibrium, as shown in Fig. 10b.

The results were interpreted that the higher availability of vacant sites on the adsorbent surface at the initial stage while by passing the time of experiments, these sites were occupied by the adsorbate molecules, and the number of vacant sites became few so the removal percent of Cr^6+^ ions and MV40 dye molecules become very slow, also it was shown that the variations of initial dye and Cr^6+^ ion concentrations did not significantly affect the removal rate to reach its equilibrium state.

Finally, we concluded that the maximum removal % of Cr^6+^ solutions was 98.60% after 180 min and the initial Cr^6+^ concentration was 100 mg L^–1^ using 2.0 g L^–1^ of CAC-IO adsorbent concentration, while 99.92% was the highest dye removal percent after 180 min using only 1.0 g L^–1^ of CAC-IO and 100 mg L^–1^ of initial dye concentration.

### Adsorption isotherms

**Figure 11:**
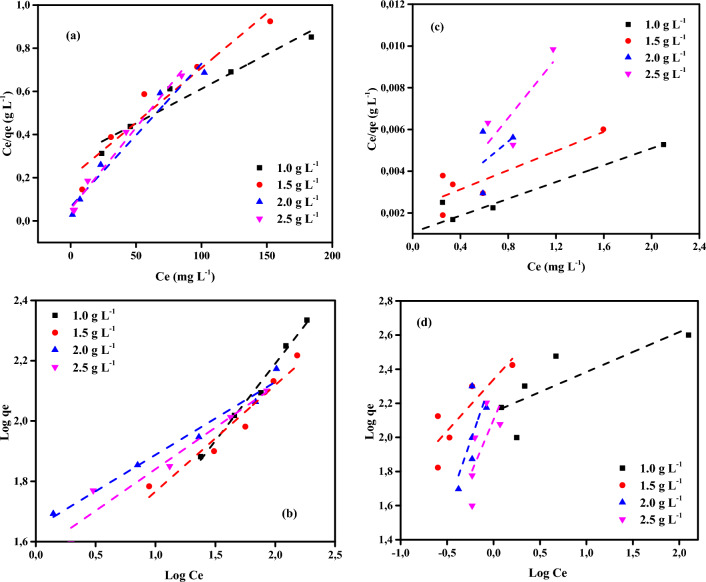
Langmuir (**a**) Freundlich (**b**) isotherm profiles for Cr^6+^ ions and Langmuir (**c**) Freundlich (**d**) isotherm profiles MV40 dye of initial concentration (100–400 mg L^–1^) on CAC-IO doses (1.00–2.50 g L^−1^) at 25 ± 2 °C, contact time: 180 min).

As indicated in Fig. 11, Langmuir and Freundlich isotherms were examined for the distinct adsorption of Cr^6+^ ions and MV40 dye on CAC-IO nanocomposites. The adsorption isotherm data of Langmuir and Freundlich models obtained using Langmuir and Freundlich models are shown in Table 3. The interaction between adsorbates and adsorbents is represented by the properties of adsorption and the parameters of each isotherm model; this information reveals the nature of the interaction^76^.

**Table 3:** Adsorption isotherm data for Cr^6+^ ions adsorption on CAC-IO nanocomposite at room temperature.

Adsorption ısotherm model	Parameters	Commercial activated carbon-iron oxide nanocomposites((CAC-IO) (g L^–1^)			
		1.0	1.5	2.0	2.5
Langmuir	R^2^	0.945	0.933	0.964	0.986
	*Q*_*m*_ (mgg^−1^)	312.50	196.08	151.52	133.33
	*K*_*a*_	0.01	0.03	0.10	0.14
Freundlich	R^2^	0.988	0.987	0.976	1.000
	*1/n*	0.51	0.44	0.24	0.31
	*K*_*F*_ (mg^1−1/n^L^1/n^g^−1^)	14.65	17.31	44.32	31.46

The Langmuir model assumed that the adsorption was monolayer on a homogeneous adsorbent surface, that there was no interaction between the molecules that were adsorbed, and that the transmigration of the molecules that were adsorbed on the adsorbent surface was not permitted^77^. The Langmuir linear Eq. (4) can be expressed as follows (Eq. (4)):

4 $$ C_{e} /q_{e} = {1}/K_{a} Q_{m} + { 1}/Q_{m} \times C_{e} , $$

where *C*_*e*_ is the concentration of adsorbate in solution (mg L^–1^) at equilibrium, *q*_*e*_ is the adsorption capacity at equilibrium in mg g^−1^, *K*_*a*_ is constant related to free energy of adsorption (L mg^–1^), and *Q*_*m*_ is the maximum adsorption capacity at monolayer coverage in mg g^–1^. An empirical linear equation of Freundlich Isotherm assumed that the adsorbent surface was heterogeneous; the equation was expressed as shown in Eq. (5):

5 $$ {\text{Ln}}q_{e} = {\text{ln}}K_{f} + {1}/n{\text{ln}}C_{e} , $$

where *K*_*f*_ (mg^1–1/n^ g^-1^ L^1/n^) and *n* are the Freundlich constants, they indicate the adsorption capacity and intensity of adsorption, respectively. The values of 1/*n* in Tables 3, 4 are greater than zero and lower than 1, (0 < 1/*n* < 1) the adsorption is favorable^78^.

**Table 4:** Adsorption isotherm data for MV40 dye adsorption on CAC-IO nanocomposite at room temperature.

Adsorption ısotherm model	Parameters	Commercial activated carbon-iron oxide nanocomposites((CAC-IO) (g L^−1^)			
		1.0	1.5	2.0	2.5
Langmuir	R^2^	0.984	0.986	0.958	0.960
	*Q*_*m*_ (mg g^−1^)	476.2	625.0	833.3	555.6
	*K*_*a*_	2.63	0.46	0.15	0.23
Freundlich	R^2^	0.983	0.979	0.995	0.983
	*1/n*	0.43	0.78	0.88	1.04
	*K*_*F*_ (mg^1−1/n^L^1/n^g^−1^)	285.10	200.26	107.57	100.02

The isotherm parameters obtained from both models due to Cr^6+^ ions adsorption on CAC-IO are listed in Table 3. It showed that the Cr^6+^ ions adsorption was best fitted by the Freundlich model as shown in Fig. 11a,b. The separation factor *R*_*L*_ was calculated by Eq. (6).

6 $$ R_{L} = {1}/{1} + K_{a} C_{o} . $$

The separation factor value (*R*_*L*_) determined the favorability of the adsorption process. It ranged from 0.02 to 0.48, so 0 < *R*_*L*_ < 1, this indicated that the adsorption of Cr^6+^ ions on the CAC-IO nanocomposite surface was favorable. The maximum adsorption capacity *Q*_*max*_ was 312.5 mg g^–1^ at 1.0 g L^–1^ of CAC-IO.

The experimental results were fitted to both isotherm models (Langmuir and Freundlich) in the adsorption of MV40 dye on CAC-IO, as shown in Fig. 11c,d. The maximum adsorption capacity (*Q*_*m*_) for the Langmuir model was 833.3 mg g^–1^, as shown in Table 4 and the correlation coefficients *R*^2^ obtained from that model varied from 0.958 to 0.986, while those obtained from the Freundlich model ranged from 0.979 to 0.995. Due to the proximity of *R*^2^ to 1, these results showed that the adsorption process was fit for both models, however, the Freundich model was better matched than the Langmuir model. Table 4 also showed that the adsorption process of dye on CAC-IO adsorbent was favorable due to 1/*n* values being lower than 1 as discussed before. The separation factors *R*_*L*_ were ranged from 0.001 to 0.063. These results indicated that the MV40 dye adsorption on CAC-IO was multilayer.

### Adsortion kinetic studies

**Figure 12:**
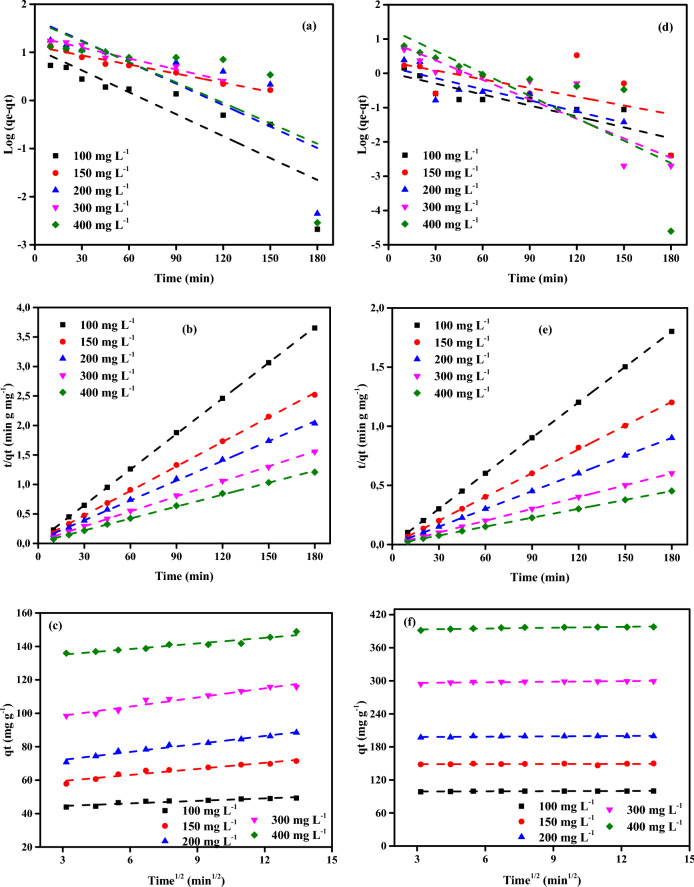
The plot of PFO (**a**) PSO (**b**) IPDM (**c**) of adsorption of Cr^6+^ ions and PFO (**d**) PSO (**e**) IPDM (**f**) of adsorption of MV40 dye by CAC-IO adsorbent (Initial concentration = (100–400 mg L^-1^), Adsorbent dose = (2.0 g L^−1^ for Cr^6+^ ions and 1.0 g L^−1^ for MV40 dye), Temperature = 25 ± 2 °C).

Three kinetic models, such as the pseudo-first-order (PFO), pseudo-second-order (PSO), and Intraprticle Diffusion (IPDM) models, were used to study the adsorption kinetic data. The rate expression of Lagergren indicated PFOas shown in Eq. (7)^79,80^:

7 $${\text{log}}({q}_{e}-{q}_{t})=\mathrm{log }({q}_{e})-\frac{{k}_{1}}{2.303}t$$

where *q*_*t*_ (mg g^−1^) is the amount of adsorbed Cr^6+^ ions on CAC-IO adsorbent in time *t* and *k*_*1*_, (min^−1^), is the first-order rate constant, *q*_*e*_ is the adsorption uptake at equilibrium. The straight line was obtained representing, log (*q*_*e*_ − *q*_*t*_) as the y-axis and *t* as the x-axis (Fig. 12a–d). The *q*_*e*_ and *k*_*1*_ shown in Tables 5 and 6 were determined from the intercept and slope of the plot, respectively. The linear PSO was used^79,80^ as in Eq. (8):

8 $$(\frac{t}{{q}_{t}})=\frac{1}{{k}_{2}{q}_{e}^{2}}+\frac{1}{{q}_{e}} \left(t\right),$$

where *k*_*2*_ (g mg^–1^) (min^–1^) is the pseudo-second-order rate constant. From the slope of the straight line *t*/*q*_*t*_ vs. *t* plot, as shown in Fig. 12b–e, we can obtain qe while *K*_2_ obtained from its intercept.

**Table 5:** Comparison of the first- and second-order adsorption rate constants and calculated and experimental *q*_*e*_ values for various initial Cr^6+^ ions and CAC-IO concentrations.

Parameter			Pseudo-first-order			Pseudo-second-order			
CAC-IO (g L^−1^)	Cr^6+^ (mg L^−1^)	*q*_*e*_ (exp.)	*q*_*e*_ (calc.)	*k*_*1*_ × 10^3^	R^2^	*q*_*e*_ (calc.)	*k*_*2*_ × 10^3^	*h*	R^2^
1.0	100	76.20	15.38	13.130	0.954	76.92	2.70	15.95	0.999
	150	104.36	24.01	11.520	0.938	105.26	1.73	19.16	0.998
	200	124.10	28.32	17.500	0.984	125.00	1.61	25.19	0.998
	300	177.48	43.28	21.650	0.955	166.67	0.60	16.78	0.982
	400	216.05	8.26	1.380	0.830	212.77	3.74	169.49	0.999
1.5	100	60.77	15.88	12.900	0.870	61.35	2.43	9.16	0.998
	150	79.45	15.81	16.350	0.927	80.00	3.09	19.76	0.999
	200	95.84	28.33	17.040	0.910	98.04	1.46	14.03	0.998
	300	135.55	19.18	11.980	0.986	135.14	2.20	40.16	0.999
	400	165.00	18.08	10.360	0.815	166.67	1.93	53.48	0.999
2.0	100	49.30	5.93	19.580	0.964	49.75	9.04	22.37	1.000
	150	71.45	13.31	14.510	0.985	71.94	3.26	16.89	1.000
	200	88.49	57.10	50.440	0.961	89.29	2.19	17.45	0.999
	300	115.77	21.62	18.190	0.974	117.65	2.10	29.07	1.000
	400	148.89	54.09	52.050	0.945	149.25	2.55	56.82	0.999
2.5	100	39.21	2.57	20.270	0.948	39.37	21.43	33.22	1.000
	150	58.79	10.10	22.110	0.972	59.52	5.68	20.12	1.000
	200	70.79	14.98	13.820	0.980	71.43	2.73	13.95	0.999
	300	103.04	18.16	12.670	0.923	104.17	2.06	22.37	0.999
	400	126.07	18.87	14.510	0.953	126.58	2.35	37.59	0.999

**Table 6:** Comparison of the first- and second-order adsorption rate constants and calculated and experimental qe values for various initial MV40 dye solutions and CAC-IO concentrations.

Parameter			Pseudo-first-order			Pseudo-second-order			
CAC-IO (g L^−1^)	MV40 (mg L^−1^)	*q*_*e*_ (exp.)	*q*_*e*_ (calc.)	*k*_*1*_ × 10^3^	R^2^	*q*_*e*_ (calc.)	*k*_*2*_ × 10^3^	*h*	R^2^
1.0	100	99.92	1.05	24.410	0.792	100.00	71.43	714.29	1.000
	150	149.66	1.83	18.190	0.978	149.25	29.93	666.67	1.000
	200	199.58	8.64	87.510	0.846	200.00	41.67	1666.67	1.000
	300	299.33	8.83	43.760	0.826	303.03	18.15	1666.70	1.000
	400	397.90	20.39	50.210	0.651	400.00	12.50	2000.00	1.000
1.5	100	66.50	1.55	60.570	0.903	66.67	48.91	217.39	1.000
	150	99.72	121.41	275.440	0.931	100.00	83.33	833.33	1.000
	200	132.94	4.32	27.640	0.837	133.33	46.88	833.33	1.000
	300	199.44	1.76	81.760	0.691	200.00	62.50	2500.00	1.000
	400	265.60	3.08	24.640	0.981	263.16	24.07	1666.67	1.000
2.0	100	49.79	6.00	195.520	0.696	49.75	577.16	1428.60	1.000
	150	74.66	1.50	95.570	0.989	74.63	299.27	1666.70	1.000
	200	99.54	1.65	32.700	0.661	100.00	37.04	370.37	1.000
	300	149.33	2.01	44.220	0.933	149.25	56.11	1250.00	1.000
	400	198.95	67.44	276.820	0.874	200.00	125.00	5000.00	1.000
2.5	100	39.76	0.27	73.010	1.000	39.84	1260.00	2000.00	1.000
	150	59.76	0.30	26.250	0.997	59.88	253.54	909.09	1.000
	200	79.70	2.22	110.770	0.978	80.00	142.05	909.09	1.000
	300	119.53	0.66	82.910	0.989	119.05	352.80	5000.00	1.000
	400	159.36	1.42	16.200	0.832	158.73	33.08	833.33	1.000

The kinetic parameter values of Cr^6+^ ions and MV40 dye adsorption on CAC-IO adsorbent were summarised in Tables 5, 6 and 7 separately. It showed that the adsorption process follows the PSO model according to correlation coefficient (*R*^2^) from 0.982 to 1.00 in the case of Cr^6+^ ions adsorption and *R*^2^ = 1 for MV40 dye adsorption and closeness of the calculated equilibrium adsorption capacity (*q*_e_)_calc_ to those obtained from the experimental value (*q*_e_)_exp_. However, *R*^2^ values for the PFO model are not satisfactory. So, the PSO adsorption model is more confirmed for an explanation of the adsorption kinetics of Cr^6+^ ions and MV40 dye by CAC-IO nanomagnetic adsorbent separately. These results were interpreted that the adsorption process was chemisorption^81^. Chemisorption is the sharing or exchanging of electrons between the adsorbate and the active sites on the adsorbent^81^.

**Table 7:** IPDM results of adsorption of Cr^6+^ ions and MV40 dye by CAC-IO adsorbent (Initial concentration = (100–400 mg L^–1^), adsorbent doses = (1.0–2.5 g L^−1^), Temp. = (25 °C)).

Parameter		Cr^6+^ ions			MV40 dye		
CAC-IO (g·L^−1^)	Pollutant (mg L^−1^)	*К*_*dif*_	*C*	R^2^	*К*_*dif*_	*C*	R^2^
1.00	100	1.440	57.51	0.923	0.111	98.57	0.720
	150	2.170	75.57	0.895	0.163	147.74	0.910
	200	2.070	96.45	0.951	0.208	197.10	0.647
	300	4.440	119.48	0.995	0.416	294.36	0.808
	400	3.460	197.11	1.000	0.391	393.11	0.816
1.50	100	1.050	45.94	0.967	0.147	64.91	0.555
	150	1.320	62.59	0.917	0.035	99.35	0.719
	200	2.010	68.91	0.993	0.481	129.10	0.672
	300	1.550	113.84	0.978	0.319	196.86	0.758
	400	1.880	140.25	0.984	0.273	262.42	0.765
2.00	100	0.500	43.09	0.884	0.015	49.62	0.602
	150	1.210	55.89	0.937	0.038	74.25	0.530
	200	1.590	67.39	0.975	0.046	98.87	0.625
	300	1.820	93.08	0.951	0.115	148.02	0.782
	400	1.120	131.73	0.912	0.097	197.87	0.572
2.50	100	0.270	36.06	0.804	0.011	39.65	0.601
	150	0.800	49.09	0.903	0.023	59.50	0.856
	200	1.270	53.91	0.975	0.046	79.18	0.584
	300	1.640	81.18	0.923	0.024	119.27	0.502
	400	1.360	107.08	0.984	0.173	157.30	0.845

To interpret the diffusion mechanism, the experimental results were analyzed and fitted to the intraparticle diffusion model (IPDM) which is expressed by the following Eq. (9):

9 $$ q_{t} = K_{diff} t^{{0.{5}}} + C, $$

where *K*_*diff*_ is the intraparticle rate constant (mg g^−1^ min^0.5^) and *C* is an intercept (mg g^−1^) which indicates the boundary layer effect. Figure 12c–f shows a linear plot of *q*_*t*_ vs *t*^0.5^, these figures showed that these parameters increased by increasing the initial concentration of Cr^6+^ ion solutions, and there was an increase of *C* due to the increase of the thickness of the boundary layer. It was seen that the linear plot didn’t pass through the origin, these indicate that intraparticle diffusion was not only the rate-determining step^82^.

The IPDM was also tested on the adsorption of the MV40 dye on CAC-IO nanocomposite, the *q*_*t*_ vs *t*^0.5^ plot was drawn as shown in Fig. 12f, and similar behavior was obtained as in the Cr^6+^ ions adsorption process in Fig. 12c, none of the lines didn’t pass through the origin and the intercepts *C* increased by increasing the initial concentrations of dye solutions from 100 to 400 mg L^–1^ at each adsorbent dose as shown in Table 7. It was concluded that the IPDM was not the only rate-controlling step as discussed previously.

### Comparison of results with reported literature

Table 8 shows some of the previous literature done for removing Cr^6+^ ions from aquatic media and dyes. The maximum adsorption capacity was recorded in this Table at a certain temperature, it was found that CAC-IO nano adsorbent has the greatest *Q*_*max*_ recorded more than the mentioned literature at room temperature. These values were 312.5 and 833.3 mg g^–1^ for Cr^6+^ ions and MV40 dye removal at fixed 1.0 g L^–1^ of nano adsorbent concentration, respectively. From this comparison, it is obvious that the CAC-IO nanocomposite prepared from CAC was an excellent adsorbent for removing Cr^6+^ ions and MV40 dye from aqueous solutions.

**Table 8:** A comparison of the highest pollutant removal capabilities of some adsorbents.

Name of adsorbent	Pollutant	*Q*_*m*_ (mg g^−1^)	Ref
Wheat straw and *E. adenophorum*	Cr^6+^	88.57	^83^
Magnetite nanoparticles	Cr^6+^	34.9	^84^
Rice husk-derived magnetic sorbent (RHC-Mag-2)	Cr^6+^	157.7	^85^
Active carbon derived from *Lantana Camara* Plant	Cr^6+^	26.25	^86^
*Pterocladia capillacea* red algae magnetic activated carbon	Cr^6+^	151.52	^10^
	Mordant Violet 40	303.03	
Activated carbon from mango kernel	Cr^6+^	7.80	^87^
CAC-IO nanocomposite	Cr^6+^	312.50	This study
CAC-IO nanocomposite	Mordant Violet 40	833.30	This study

### Regeneration of MG-OPAC

**Figure 13:**
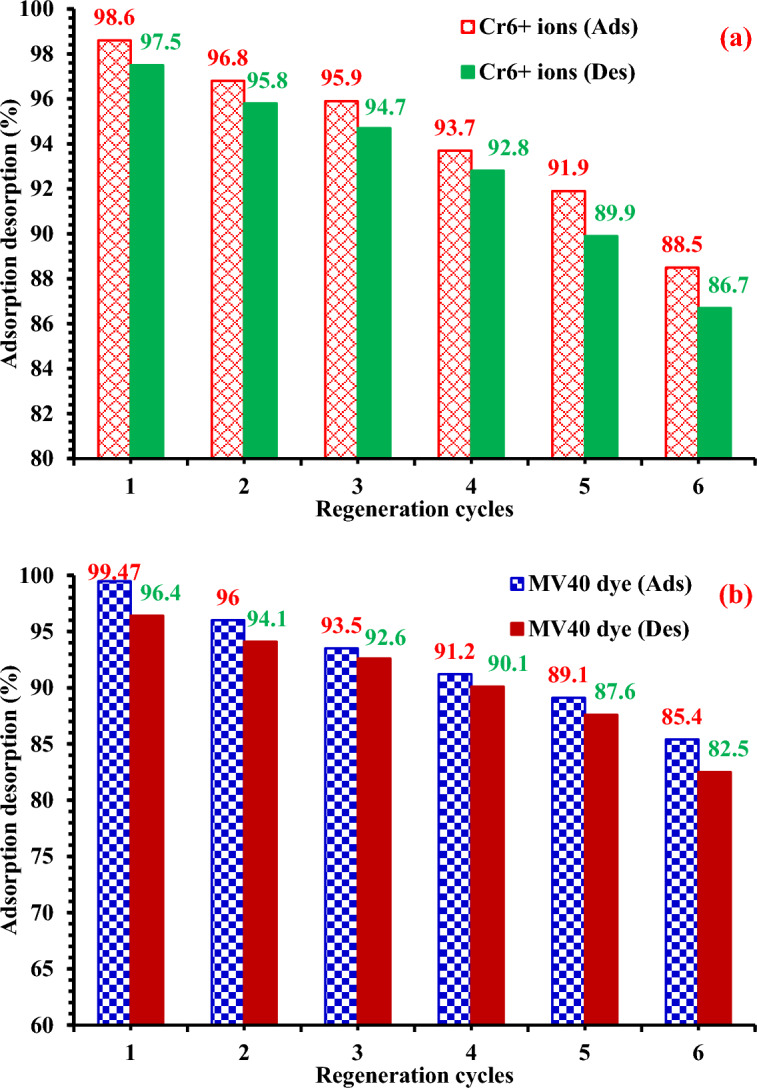
(**a**) Cr^6+^ ions desorption% from CAC-IO (2.0 g L^–1^) and the regenerated CAC-IO was used to promote Cr^6+^ ions (100 mg L^–1^) adsorption cycles using pH = 1.0, (**b**) MV40 dye was desorption% from CAC-IO (1.0 g L^–1^) and the regenerated CAC-IO was used to promote MV40 dye (400 g L^–1^) adsorption cycles using pH = 2.07.

To test the viability and reusability of CAC-IO as an adsorbent, desorption tests of the Cr6 + ions and MV40 dye from the CAC-IO adsorbent were carried out by 0.1 M NaOH and HCl as elution media. With increasing regeneration cycles in this situation, the desorption percentage dropped (Fig. 13a,b). The regenerated CAC-IO was used in six successive adsorption/desorption cycles for the two pollutants with Slightly better for Cr^6+^ ions. The amount of adsorption that was offered remained constant during the cycles; however, after six regenerations, the adsorption capacity of Cr^6+^ ions had decreased by 10.1%, while the desorption capacity decreased by 10.8% after six desorption cycles. On the other hand, After six regenerations, the adsorption capacity of MV40 dye had decreased by 14.1%, while the desorption capacity decreased by 13.9% after six desorption cycles. It implies that it might be employed as a long-lasting Cr^6+^ ions and MV40 dye adsorption process (Fig. 13a,b).

### Adsorption mechanism of Cr^6+^ ions and MV40 dye ions by CAC-IO

**Figure 14:**
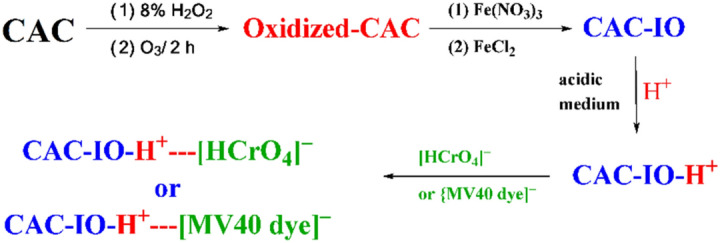
Probable mechanism for the Cr^6+^ and MV40 dye ions adsorption onto the CAC-IO in acidic medium.

In the case of Cr^6+^ ions and MV40 dye, the probable adsorption mechanism onto magnetic commercial activated carbon (CAC-IO) in acidic medium was explained in Fig. 14. The activated carbon possesses numerous surface functional groups such as hydroxyl (-OH), carboxyl (-COOH), and other polar moieties. These functional groups play a crucial role in attracting and holding the pollutant molecules. Cr^6+^ ions and MV40 dye are likely to have charged particles, as many pollutants are ionic or polar in nature. The activated carbon, being a porous material, has a large surface area with a distribution of positive and negative sites. After the oxidation of the CAC, many functional groups were formed on the adsorbent (CAC) surface like allene C=C=C, ketamine C=C=N, hydroxyl O–H, and C–N groups. CAC, with its graphitic structure, can form π-π interactions with these aromatic rings. This type of interaction enhances the adsorption capacity, especially when the activated carbon is magnetic. The mechanism of the removal of Cr^6+^ ions and MV40 dye in an acidic medium may be achieved via physical interaction due to electrostatic interaction between the positive hydrogen ions in the bulk solution and the nitrogen and oxygen functional groups on the CAC-IO surface, then surface charge became positive; subsequently electrostatic interaction was occurred between the positively charged surface and the predominant pollutant anionic species ([HCrO_4_]^–^ and [MV40]^–^ dye).

## Conclusion

Iron oxide nanocomposite (CAC-IO) was prepared from commercial activated carbon (CAC) by co-precipitation method using different iron salts and different base solutions. The Cr^6+^ ions removal % from its solution by CAC-IO adsorbent was 98.60% at solution pH 1.6 and adsorbent concentrations 2.0 g L^–1^. The removal % of MV40 dye was 99.92% by CAC-IO, at pH of dye solutions = 2.07, and adsorbent concentrations of 1.0 g L^−1^ of CAC-IO at 100 mg L^−1^ of initial dye concentration. The removal percentage of Cr^6+^ ions and MV40 dye was higher in acidic solutions than in basic solutions. CAC-IO nanocomposite has 7.4130 emu g^–1^ magnetization saturation. *Q*_*max*_ of Cr^6+^ ions on CAC-IO was 312.50 mg g^−1^ at 1.0 g L^–1^, while in the case of MV40 dye, it was 833.3 mg g^–1^ at 2.0 g L^–1^ adsorbent concentration. Freundlich model was the most fitted on MV40 dye adsorption using CAC-IO; also it was the best fitted model in Cr^6+^ ions adsorption on CAC-IO. CAC-IO nanocomposite can be separated from aqueous media after treatment and adsorption process by a magnet. An encouraging development in water treatment technology is the incorporation of magnetic characteristics into activated carbon usch as preparation of CAC-IO. The combination of magnetic separation and adsorption offers a flexible and effective way to deal with problems related to water quality, all the while enhancing the overall sustainability of water treatment procedures. Pursuing cleaner and more sustainable water resources is the goal of ongoing research and development in this subject, which aims to broaden and improve the uses of CAC-IO. The prepared Iron oxide nanocomposite CAC-IO can be used for the adsorption of Cr^6+^ ions and MV40 dye from aqueous media. The regeneration of CAC-IO was effective up to six cycles, which explains the possibility of using the prepared CAC-IO in treating industrial wastewater with high effectiveness, which may lead to reducing the cost of treating industrial wastewater.

## Data Availability

The data presented in this study are available on the request from the corresponding author.
